# REVERSE phenotyping—Can the phenotype *following constitutive Tph2 gene inactivation in mice* be transferred to children and adolescents with and without adhd?

**DOI:** 10.1002/brb3.2054

**Published:** 2021-02-01

**Authors:** Atae Akhrif, Arunima Roy, Katharina Peters, Klaus‐Peter Lesch, Marcel Romanos, Angelika Schmitt‐Böhrer, Susanne Neufang

**Affiliations:** ^1^ Department of Child and Adolescent Psychiatry Center of Mental Health University of Würzburg Würzburg Germany; ^2^ Comparative Psychology Institute of Experimental Psychology Heinrich‐Heine University of Düsseldorf Düsseldorf Germany; ^3^ The Royal’s Institute of Mental Health Research University of Ottawa Ottawa Canada; ^4^ Division of Molecular Psychiatry Center of Mental Health University of Würzburg Würzburg Germany; ^5^ Laboratory of Psychiatric Neurobiology Institute of Molecular Medicine Sechenov First Moscow State Medical University Moscow Russia; ^6^ Department of Psychiatry and Neuropsychology School for Mental Health and Neuroscience Maastricht University Maastricht The Netherlands; ^7^ Department of Psychiatry, Psychosomatics and Psychotherapy Center of Mental Health University of Würzburg Würzburg Germany; ^8^ Department of Psychiatry and Psychotherapy Medical Faculty Heinrich Heine University of Düsseldorf Düsseldorf Germany

**Keywords:** aggression, anxiety, attention‐deficit/hyperactivity disorder (ADHD), impulsivity, *TPH*2 *G‐703T* (rs4570625) polymorphism, *Tph2^‐/‐^* mouse, tryptophan hydroxylase‐2 (TPH2)

## Abstract

**Introduction:**

Experimental models of neuropsychiatric disorders, for example, ADHD, are used to mimic specific phenotypic traits of a complex human disorder. However, it remains unresolved to what extent the animal phenotype reflects the specific human trait. The null mutant mouse of the serotonin‐synthesizing tryptophan hydroxylase‐2 (*Tph2* ^‐/‐^) gene has been proposed as experimental model for ADHD with high face validity for impulsive, aggressive, and anxious behaviors. To validate this ADHD‐like model, we examined the *Tph2^‐/‐^ phenotype* in humans when considering allelic variation of TPH2 function (“reverse phenotyping”).

**Methods:**

58 participants (6 females, 8–18 years) were examined, of whom 32 were diagnosed with ADHD. All participants were phenotyped for impulsivity, aggression, and anxiety using questionnaires, behavioral tests, and MRI scanning while performing the 4‐choice serial reaction time task. Additionally, participants were genotyped for the *TPH*2 *G‐703T* (rs4570625) polymorphism. To analyze the relation between *TPH*2 *G‐703T* variants and the impulsive/aggressive/anxious phenotype, mediation analyses were performed using behavioral and MRI data as potential mediators.

**Results:**

We found that the relation between *TPH*2 *G‐703T* and aggression as part of the *reverse Tph2^‐^/^‐^ phenotype* was mediated by structure and function of the right middle and inferior frontal gyrus.

**Conclusion:**

At the example of trait aggression, our results support the assumption that the *Tph2* null mutant mouse reflects the *TPH*2 *G‐703T‐*dependent phenotype in humans. Additionally, we conclude that “reverse phenotyping” is a promising method to validate experimental models and human findings for refined analysis of disease mechanisms.

## Graphical abstract

The *Tph2^‐/‐^* mouse has been proposed as an experimental model for ADHD. Translation of an animal phenotype into humans to examine its face validity (reverse phenotyping). Phenotyping included impulsivity, aggression, and anxiety, reflecting comorbid conditions of ADHD. Use of the RDoC approach to address phenotype dimensions in an isolated manner.

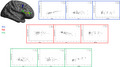

## 1 INTRODUCTION

Attention‐deficit/hyperactivity disorder (ADHD) is a highly heterogenous disorder that frequently presents with comorbidities such as oppositional defiant and conduct disorder (prevalence: 67%, 46%) and anxiety disorders (prevalence: 44%) (Steinhausen et al., 2006). Translational studies have gained importance in neuropsychiatric research, improving our understanding of mechanisms and pathways to comorbidities. In the case of the serotonin‐synthesizing tryptophan hydroxylase‐2 (TPH2) gene, the null mutant mice (*Tph2* ^‐/‐^) have been discussed as an experimental model for ADHD. Functional variants of the human *TPH*2 gene have been associated with ADHD in numerous studies (Manor et al., 2008; McKinney et al., 2008; Sheehan et al., 2005; Walitza et al., 2005), and *Tph2* ^‐/‐^ mice are characterized by increased impulsivity (Lesch & Merschdorf, 2000; Sachs et al., 2013), which is a core symptom of ADHD. However, as the *Tph2* ^‐/‐^ mouse also demonstrates increased aggression and decreased anxiety‐like behavior (Lesch et al., 2012; Waider et al., 2019), it is not known to what extent the *Tph2* ^‐/‐^ mouse reflects ADHD symptomatology. To investigate whether the alterations *following constitutive Tph2 gene inactivation in mice* can also be found in humans via the way the *TPH2* influences human (impulsive, aggressive, and anxious) behavior, we employ the “*reverse phenotyping”* approach “where phenotypes are refined based on genetic marker data” (Schulze & McMahon, 2004). Based on the assumption that the animal phenotype reflects ADHD‐associated symptoms, we examined children and adolescents with and without ADHD. Thus, in this study, the *reverse Tph2* ^‐/‐^ *phenotype* refers to the examination of the animal phenotype (i.e., altered impulsivity, aggression, and anxiety) in humans.

TPH2 catalyzes the rate‐limiting step in the biosynthesis of serotonin (Zhang et al., 2004). It is primarily expressed in the serotonergic neurons of the raphe nuclei, which project to brain regions including the hippocampus (Brivio et al., 2018; Migliarini et al., 2013; Zill et al., 2004; Zill et al., 2004), amygdala (Brown et al., 2005; Haghighi et al., 2008), the prefrontal cortex (Baehne et al., 2009; Haghighi et al., 2008), and the anterior cingulate (Canli et al., 2008). To “explore the question of what traits or neuropsychiatric disorders are attributable to TPH2 dysfunction across the life span” (Lesch et al., 2012), three different *Tph2*‐deficient mouse lines were generated close in time with a targeted inactivation of *Tph2* (Alenina et al., 2009; Gutknecht et al., 2008; Savelieva et al., 2008). Reduced *Tph2* expression is associated with an impulsivity‐, aggression‐, and anxiety‐related phenotype combined with alterations in prefrontal cortex, anterior cingulate, and amygdala (Coccaro et al., 2011; Ko et al., 2018; Lesch et al., 2012; Mark et al., 2019; Waider et al., 2019).

Altered impulsivity, aggression, and anxiety also correspond to phenotypes frequently observed in patients with ADHD, especially in the context of the abovementioned comorbidities. In humans, the influence of TPH2 is often addressed using either the tryptophan depletion approach or by stratification for the functional *TPH2 G‐703T* (rs4570625) polymorphism. In patients with ADHD and typically developing children (TDC), tryptophan deficiency has been associated with increased impulsivity (Stoltenberg et al., 2012), heightened aggression (Duke et al., 2013), and altered anxiety (Lowry et al., 2008). For example, carriers of the *TPH2* T allele (T^+^) presented increased risk of impulse control disorders (Gizer et al., 2009), disturbed affective behavior (Canli et al., 2008), and reduced response inhibition (Stoltenberg et al., 2012). Studies using tryptophan depletion on aggression in TDC and ADHD (Kotting et al., 2013; Polier et al., 2013) showed that depleted patients reacted strongest in comparison with nondepleted patients and TDC. In socially anxious T^+^ patients, an overactive presynaptic serotonergic neurotransmission in the amygdala and anterior cingulate was described (Furmark et al., 2016), and in a sample of patients with ADHD with and without comorbid anxiety, anxiety was related to a reduction in impulsivity and response inhibition deficits suggesting that a “mildly elevated trait anxiety confers a protective influence by reducing the degree of impairment seen in ADHD” (Ruf et al., 2016).

In this fMRI study, we examined children with and without ADHD. Participants were genotyped for *TPH2 G‐703T* and phenotyped according to trait impulsivity, aggression, and anxiety. In addition, structural and functional MRI was acquired using the 4‐choice serial reaction time task (4‐CSRTT) (Voon et al., 2014). Waiting impulsivity as measured via the 4‐CSRTT is one form of impulsivity, defined as the tendency to premature responding, that is, to respond before target onset. Thus, it involves the aspects of response inhibition and top‐down control, mediated by motivational aspects and reward processing (Robinson et al., 2009; Voon, 2014). The underlying brain network includes the ventromedial and dorsolateral prefrontal cortex as well as the anterior cingulate cortex representing motor or response inhibition (Mechelmans et al., 2017), the reward‐related nucleus accumbens (Neufang et al., 2016), and hippocampus and amygdala responsible for reward‐based learning (Dalley et al., 2011). We used the dimensional approach as suggested by the rDoC (https://www.nimh.nih.gov/research/research‐funded‐by‐nimh/rdoc/index.shtml), to address neuropsychiatric impairments in terms of a gradual change rather than a binary factor (ADHD versus. TDC). Thus, all subjects were examined as one sample using regression‐based statistical analyses.

The aim of the study was to examine how *TPH2 G‐703T* was related to trait impulsivity, aggression, and anxiety (i.e., the *reverse Tph2^‐/‐^ phenotype*) in children and adolescent with and without ADHD. Additionally, the question was if brain structure and function mediated this relation. Therefore, *TPH2 G‐703T* variant group comparisons were performed in a first step, to see whether *TPH2 G‐703T* directly influences phenotype variables. In a second step, mediation analyses were performed to reveal whether the influence of *TPH2 G‐703T* on phenotype variables indeed was direct or rather indirect via neural parameters.

Based on the earlier reported findings, we assumed that *TPH2 G‐703T* modulated all three phenotype traits: considering the protective effect of anxiety on ADHD, we hypothesized a positive effect on anxiety (i.e., GG > T^+^). In contrast, the influence of aggression and impulsivity was supposed to be negative, that is, T^+^ presented higher impulsive and aggressive trait scores compared with GG. However, we did not expect to find significant differences between *TPH2 G‐703T* variants on impulsivity, aggression, and anxiety (direct effect), but assumed that the relation between *TPH*2 *G‐703T* and impulsivity, aggression, and anxiety was mediated by neural structure and/or function (indirect effects), for example, of the prefrontal cortex and the amygdala.

## 2 METHODS

### 2.1 Subjects

We examined 59 subjects (6 females), comprising 32 patients with ADHD (3 females) and 26 TDC (3 females). One ADHD patient did not finish MRI scanning and was excluded from statistical analyses. Remaining 58 subjects were aged from 8 to 18 years. *M* = 13.5 ± 2.2 years showed normal physical maturation (Tanner stages: *M* = 3.1 ± 1.2), (Marshall & Tanner, 1969, 1970) and intelligence was screened via the "Culture Fair Intelligence Test" (Weiß, 2006) (*M* = 105.0 ± 14.4, range: 80–153). Healthy participants were recruited within the CRC‐TRR‐58, and patients with ADHD were recruited from in/outpatient clinics of the Department of Child and Adolescent Psychiatry Würzburg, within the research network ESCAlife. All patients were diagnosed with ADHD according to the DSM‐V by trained clinicians (Table S1). Eighteen patients were medicated with methylphenidate (9 retard, daily dosage: *M* = 30±10.8 mg), and unmedicated patients were either medication‐naïve (*n* = 8) or stopped medication use for more than a year (*n* = 6). Medicated patients underwent a washout phase of 48 hr (*N* = 11) or longer (*N* = 7, *M* = 22 ± 22.8 day) prior to scanning.

This study is in accordance with the Declaration of Helsinki in its latest version and was approved by the ethics committee of the Faculty of Medicine, University of Würzburg, Germany. Participants and their parents/legal guardians gave written consent.

### 2.2 Reverse *Tph2^‐/‐^* Phenotyping

The *reverse Tph2^‐/‐^ phenotype* was determined via the (a) “hyperactivity/impulsivity” scale of the "Diagnostic System for Mental Disorders in Children and Adolescents according to ICD‐10 and DSM‐V" (Döpfner & Görtz‐Dorten, 2017), (b) the questionnaire for aggressive behavior in children and adolescents (Goertz‐Dorten & Döpfner, 2010), and (c) the trait scale of German version of the State‐Trait Anxiety Inventory for Children (Unnewehr et al., 1992). All three instruments were administered at the examination day, and completion was supervised by an examiner.

### 2.3 Genotyping

Blood sampling and genotyping were performed in all subjects. Genomic DNA was extracted from whole‐blood samples according to a standard desalting protocol. Genotyping procedures were performed using PCR and gel electrophoresis. Genotyping for the *TPH*2 *G‐703T* (rs4570625) polymorphism was performed according to published protocols (Hahn et al., 2013). *TPH*2 *G‐703T* distribution (TT = 0, 0%; GT = 25, 43.1%; GG = 33, 56.9%; *p*(Exact) = .7845) did not deviate significantly from the expected numbers calculated according to the Hardy–Weinberg equilibrium using the program DeFinetti provided (https://wpcalc.com/en/equilibrium‐hardy‐weinberg/). Based on the findings showing that TPH2 expression is decreased in carriers of the T allele (Lin et al., 2007), we defined two groups, subjects homozygous for the *TPH*2 G allele (GG, *n* = 33) and carriers of at least one T allele (T^+^, *n* = 25).

### 2.4 Experimental paradigm

The 4‐CSRTT examines waiting impulsivity defined as the capability to inhibit a response to earn a reward. A trial begins with a short presentation of 4 boxes, which starts the waiting period. After a certain cue–target interval, the target appears as a green dot, located in one of the four boxes. Correct and quick responding was remunerated by a monetary reward. Premature responses were defined as reactions before target onset in the anticipation of reward. The task consisted of one block outside the scanner (2.5 min) and five blocks within the MR scanner (14 min) (Neufang et al., 2016).

All participants were trained outside the scanner and prior to the actual task. After training sessions, a first baseline block was conducted to determine the individual mean reaction time window (rt, M_rt_ ± 2*SD*). The baseline block consisted of 20 trials and did not include a reward. The individual rt windows were used for reward definition in all consecutive blocks, which were performed in the MR scanner. Reward was provided in terms of a win of 10 euro cents, when the answer was correct and within the individual rt window, or a win of 1 euro when subjects answered correctly and were faster than their individual rt window. Subjects encountered a loss of 1 euro when response times for correct responses were longer than the rt window. Incorrect answers were neither rewarded nor punished.

The experiment in the scanner included 5 further blocks, 4 blocks with the opportunity to earn a reward and a second baseline block without reward, with each having 20 trials. The 4 reward blocks had a hierarchical structure to increase the tendency for premature responses. In detail, in the 1st block in the scanner, the presentation duration of the target was 64 ms, and the cue–target interval was 2 s and only green, that is, correct targets were presented. In block 3, target presentation duration was decreased to 32 ms. In block 4, presentation duration remained 32 ms; in addition, cue–target interval varied from 2 s to 6.5 s. Finally in block 5, in addition to short presentation duration and varying cue–target intervals, distractor targets were presented in terms of blue or yellow dots (for further detail see (Neufang et al., 2016)). Total task duration was 14 min in the MR scanner and 2.5 min for the first baseline block.

### 2.5 Data acquisition

Scanning was performed on a 3 Tesla TIM Trio Scanner (Siemens). Whole‐brain T2*‐weighted BOLD images were recorded with a gradient echo‐planar imaging sequence (repetition time = 2000 ms, echo time = 30 ms, 36 slices, 3 mm thickness, field of view = 192 mm, flip angle = 90°, 425 volumes). In addition, an isotropic high‐resolution T1‐weighted three‐dimensional structural MR image was acquired (magnetization prepared rapid gradient echo, 176 slices, 1 × 1 × 1 mm^3^, repetition time = 2400 ms, echo time = 2.26 ms, field of view = 256 mm, flip angle = 9°).

### 2.6 fMRI processing

Data processing was performed using the Statistical Parametric Mapping Software Package (SPM12, Wellcome Department of Imaging Neuroscience, London, UK, Wellcome Trust Centre for Neuroimaging; http:// www.fil.ion.ucl.ac.uk/spm/). Data preprocessing in the native space included the steps of temporal and spatial alignment: All images were slice time‐corrected, realigned to the first functional image, and unwarped. Images were then spatially normalized into a standard stereotactic space (Montreal Neurological Institute), resampled to an isotropic voxel size of 2 × 2×2 mm^3^, and spatially smoothed with a Gaussian kernel of 8mm full width at half maximum. Statistical analysis on the individual first level (single‐subject level) was based on the general linear model (GLM) approach. Model specification included the definition of experimental condition, in our case “cue,” “target,” and “reward.” Reward trials were subdivided into “win” and “loss” trials. Break periods were defined as “rest.” In addition to the experimental conditions, nuisance regressors were specified, that is, “error trials” and “realignment parameters” (i.e., six regressors containing movement in three spatial and three rotational axes), to correct for error variance and movement artifacts. For each condition, onset times were determined from log files with onsets of the cue condition were determined at the time when the cue picture was presented. Onset times of target trials were defined in terms of the appearance of the target picture, and onset times of reward trials (win and loss) were the time points when the reward feedback picture appeared on the screen. The onsets of error trials were defined as the target onsets of incorrect trials. On the single subjects, three contrasts of interest were calculated, “cue–rest” to identify cue‐specific brain activation, “target–rest” to isolate target‐induced brain activation, and “reward” in terms of “win–loss” to identify brain activation associated with the receipt of monetary reward. Resulting contrast images entered statistical group analysis. For mediation analyses, beta values of brain areas which were significantly related to the *reverse Tph2^‐/‐^ phenotype* were extracted.

### 2.7 sMRI processing

Structural MRI data were analyzed using the FreeSurfer (versions 5.3) software (Fischl et al., 2002). Analysis and quality‐control protocols of the ENIGMA consortium (http://enigma.ini.usc.edu/protocols /imaging‐protocols) were applied including the recon‐all ‐all stream and the segmentations of 68 (34 left and 34 right) cortical gray matter regions based on the Desikan–Killiany atlas (Desikan et al., 2006). In this study, we focused on regions associated with waiting impulsivity, that is, the inferior frontal gyrus (IFG, triangular/opercular/orbital part), the middle frontal gyrus (MFG), the anterior cingulate, hippocampus, amygdala, and nucleus accumbens. Regional volume scores were corrected for global brain volume in terms of % of intracranial volume.

### 2.8 Mediation analyses

In line with earlier publications (e.g., (Bi et al., 2017)), we performed mediation analyses to address the complex mechanism how *TPH*2 *G‐703T* was related to the *reverse Tph2^‐/‐^ phenotype* and which roles brain structure and activation played. We used the Macro PROCESS for SPSS developed by Hayes (Hayes, 2017) (http://www.processmacro.org/download.html), which has been used in numerous earlier scientific reports, for example, (Kneer et al., 2020; Mertens et al., 2018). The simple mediation model includes two consequent variables; thus, two linear models are required and defined as follows:

(1) $$M=i_{M}+{\text{aX + e}}_{M}$$

(2) $$Y=i_{Y}+{\text{c'X + bM + e}}_{Y}$$

with *i*_M_ and *i*_Y_ being regression constants; e_M_ and e_Y_, error estimates of M and Y; and a, b, and c’, the regression coefficients given to the antecedent variables in the model (Figure S1 X, Y, M1, a1, b1 and c’). When using control variables (e.g., in our case diagnosis and age), the simple mediation model changes as follows:

(3) $$M=i_{1}+aX+\sum_{j‐1}^{k}\sigma C_{j}+\varepsilon_{M}$$

(4) $$Y=i_{2}+y^{\prime }X+\beta M+\sum_{j‐1}^{k}\tau_{j}C_{j}+\varepsilon_{y}$$

with y’ representing the direct effect of X on Y, αβ defining the indirect effect of X on Y as mediated via M, and the sum of y’ + αβ the total effect of X on Y.

In this study, we defined three models, one for anxiety, one for impulsivity, and one for aggression, with *TPH2 G‐703T* as independent factor (X) and the *reverse Tph2^‐/‐^ phenotype* as dependent variables (Y) and four mediator variables. Mediator variables were the two impulsivity/aggression/anxiety‐specific activation scores (Table 2) and the corresponding regional volume (i.e., impulsivity‐associated brain activation in the right IFG pars triangularis (M1) and the right MFG (M2), and the volumes of IFG_tri_ (M3) and MFG (M4); aggression‐related brain activation in the same regions and their structural volumes; and activation correlated with anxiety in the right IFG pars opercularis and the MFG, and the regional volumes of the IFG_op_ and the MFG] (Figure S1). All three models were tested twice varying between parallel (PROCESS, model 4) and serial mediation effects (PROCESS, model 6). In the case of serial mediation, the total effect of c is dissected into five different indirect effects:

(5) $$c=c^{\prime }+a_{1}b_{1}+a_{2}b_{2}++a_{3}b_{3}+a_{4}b_{4}$$

**TABLE 2:** Multiple regressions of the *reversed Tph2^‐/‐^ phenotype* on brain activation using multiple regression models (independent factors: impulsivity, aggression, anxiety)

Task condition	Regressor	k	x	y	z	Z	region
Cue	Impulsivity^(+)^	15	−46	6	16	3.2	Left IFG_op_
Cue	Aggression^(+)^	32	46	12	10	3.0	Right IFG_op_
Target	Anxiety^(+)^	43	−44	22	6	3.5	Right IFG_op_
Reward	Impulsivity^(+)^	169	40	50	8	3.6	Right MFG
			38	34	8	3.4	Right IFG_tri_
Reward	Aggression^(‐)^	71	40	36	8	3.1	Right IFG_tri_
			44	48	4	3.1	Right MFG
Reward	Anxiety^(+)^	20	38	22	54	3.4	Right MFG

^(+)^: positive correlation, ^(‐)^: negative correlation, IFG_op_, opercular part of the inferior frontal gyrus, IFG_tri_, triangular part of the inferior frontal gyrus, MFG, middle frontal gyrus, FDR correction *p*<.05 on voxel level.

Statistical significance of direct effects was reached when *p *< .05, FDR‐corrected for 15 comparisons (for a detailed description of direct effects, Table 3). Indirect effects were tested for significance using bootstrapping (no of bootstrap samples: 10,000). In this case, the test for significance was one‐sided assuming that indirect effects (i.e., αβ as the indirect effect of X on Y, mediated via M) were > 0. Therefore, bootstrapped confidence intervals of 95% were defined. If confidence intervals did not include 0, the indirect effect was considered as significant.

**TABLE 3:** Mediation model with the independent factor X = *TPH*2 *G‐703T*, the dependent variable Y = aggression, potentially mediating variables M1 = aggression‐related activation in the right IFG_tri_, M2 = aggression‐associated activation in the MFG, M3 = right IFG_tri_ volume, and M4 = right MFG volume, as well as nuisance variables: age and diagnostic group

Variable 1	Variable 2	Path	Coeff	*SE*	T, *p*	LLCI	ULCI
*Direct effects*							
TPH2(X)	act_rIFGtri_(M1)	a1	1.60	0.66	2.4, *p*=.02	0.27	2.92
	act_rMFG_(M2)	a2	−0.10	0.73	0.1, *p*=.89	−1.56	1.36
	vol_rIFGtri_(M3)	a3	0.01	0.01	0.2, *p*=.90	−0.03	0.03
	vol_rMFG_(M4)	a4	0.06	0.04	1.4, *p*=.16	−0.02	0.14
	aggression(Y)	c’	8.13	6.65	1.2, *p*=.23	−5.24	21.50
act_rIFGtri_(M1)	act_rMFG_(M2)	d21	0.98	0.14	6.8, *p*=.00	0.69	1.26
	vol_rIFGtri_(M3)	d31	−0.01	0.01	0.3, *p*=.78	−0.01	0.01
	vol_rMFG_(M4)	d41	−0.02	0.01	1.9, *p*=.06	−0.04	0.01
	aggression(Y)	b1	−1.26	1.83	0.7, *p*=.49	−4.94	2.41
act_rMFG_(M2)	vol_rIFGtri_(M3)	d32	0.01	0.01	0.1, *p*=.95	−0.01	0.01
	vol_rMFG_(M4)	d42	0.02	0.01	2.2, *p*=.04	0.01	0.03
	aggression(Y)	b2	−1.70	1.30	1.3, *p*=.20	−4.10	0.91
vol_rIFGtri_(M3)	vol_rMFG_(M4)	d34	0.58	0.41	1.4, *p*=.16	−0.24	1.40
	aggression(Y)	b3	28.99	67.56	0.4, *p*=.67	−106.79	164.76
vol_rMFG_(M4)	aggression(Y)	b4	39.25	23.04	1.7, *p*=.10	−7.05	85.54

		Effect	bootSE^a^	bootLLCI^a^	bootULCI^a^
*Indirect effects (Ind)*					
Ind1:	XM1Y	−2.02	3.27	−9.93	3.27
Ind2:	XM1M2Y	−2.64	2.58	−9.62	1.03
Ind3:	XM1M3Y	−0.05	0.58	−1.72	0.82
**Ind4:**	**XM1M4Y**	**−1.31**	**1.35**	**−6.27**	**−0.02**
Ind5	XM1M2M3Y	0.01	0.31	−0.56	0.65
**Ind6:**	**XM1M2M4Y**	**1.01**	0.**97**	0.**01**	**4.50**
Ind7:	XM1M3M4Y	−0.04	0.25	−0.95	0.21
Ind8:	XM1M2M3M4Y	0.01	0.12	−0.15	0.41
Ind9:	XM2Y	0.17	1.62	−2.14	4.88
Ind10:	XM2M3Y	−0.01	0.13	−0.31	0.21
Ind11:	XM2M4Y	−0.07	0.60	−1.87	0.83
Ind12:	XM2M3M4Y	−0.01	0.06	−0.14	0.09
Ind13:	XM3Y	0.05	0.91	−1.29	2.67
Ind14:	XM3M4Y	0.04	0.42	−0.54	1.34
Ind15:	XM4Y	2.22	2.48	−0.63	9.70

Note

Abbreviations: act_rIFGtri_, aggression‐related activation in the right IFG_tri_; act_rMFG_, aggression‐associated activation in the MFG; vol_rIFGtri_, right IFG_tri_ volume; vol_rMFG_, right MFG volume.

^a^ no of bootstrap samples: 10,000; level of confidence for all confidence intervals: 95%; FDR correction for 15 comparisons revealed a q*=0.003; **bold**: significant indirect effect as CI does not include 0.

### 2.9 Statistical analysis

1. In a first step, group comparisons were performed on phenotype scores using two‐sample tests with the independent factor *TPH2 G‐703T* (GG versus. T^+^) and phenotype‐related trait dimensions (impulsivity, aggression, and anxiety) as dependent variables.
2. To address the interaction between *TPH2 G‐703T* genotype, the *reverse Tph2^‐/‐^ phenotype,* and the waiting impulsivity network, mediation analyses were defined. To do so, waiting impulsivity parameters which were influenced by the *reverse Tph2^‐/‐^ phenotype* had to be identified in a pre‐analysis using multiple regressions (2a) with phenotype scores as independent factors, (a) behavioral performance (# premature responses, accuracy, rt), (b) regional brain volumes, and (c) neural activation (contrast images for cue, target, and reward) as dependent variables. Age and diagnostic group (ADHD versus. TDC) were included as nuisance variables. (2b) Finally, mediation analyses were performed We used PROCESS model 4 (assuming mediators operate in parallel) and PROCESS model 6 (assuming that mediators operate in serial) as suggested by Hayes (Hayes, 2017) (Figure S1).

## 3 RESULTS

There were no significant a priori differences between *TPH*2 *G‐703T* variants in behavioral performance and regional brain volume (Table 1) (for differences between diagnostic groups Table S1). Across all subjects, task accuracy and rt varied with age: accuracy increased and rt decreased with age. Developmental changes were comparable between *TPH*2 *G‐703T* variants (accuracy*age: R_GG_ = 0.461, *p *= .007, R_T+_ = 0.446, *p *= .025; Z _GGvs.T+_ **= 0.1, *p *= .464; rt*age: R_GG_ **= −0.437, *p *= .029, R_T+_ **= −0.687, *p *= .000; Z_GGvs.T+_ **= 1.7, *p *= .075).

**TABLE 1:** A priori group differences between *TPH*2 *G‐703T* polymorphisms using two‐sample *t* tests (*GG* versus *T^+^*)

	GG	T^+^	Statistics
Sample characteristics			
Diagnostic group (ADHD/TDC)	14/18	17/8	*Χ* ^2^ = 2.9, *p*=.07
Sex(male/female)	30/3	22/3	*Χ* ^2^ = 0.1, *p*=.52
Age	13.9 ± 2.2	13.1 ± 2.3	T_(56,2)_=1.3, *p*=.18
IQ	104.4 ± 16.7	105.9 ± 10.9	T_(56,2)_=0.4, *p*=.69
Tanner stages	3.2.±1.1	2.8 ± 1.3	T_(56,2)_=1.2, *p*=.22
Reversed Tph2^‐/‐^ phenotype			
Impulsivity (FBB_Imp)	0.5 ± 0.6	0.9 ± 0.9	T_(56,2)_=2.0, *p*=.05
Aggression (FAVK)	33.4 ± 23.5	45.2 ± 29.7	T_(56,2)_=1.7, *p*=.10
Anxiety (STAIC‐T)	32.6 ± 7.2	32.7 ± 9.2	T_(56,2)_=0.11, *p*=.97
A priori group differences			
Behavioral performance			
Premature responses	2.8 ± 1.7	2.6 ± 1.8	T_(56,2)_=0.3, *p*=.80
Accuracy [%]	79.8 ± 15.6	81.1 ± 14.4	T_(56,2)_=0.3, *p*=.75
Reaction times [ms]	439 ± 93	464 ± 101	T_(56,2)_=1.0, *p*=.32
Regional volumes			
Left HC	0.27 ± 0.08	0.28 ± 0.07	T_(56,2)_=0.7, *p*=.51
Left AMY	0.12 ± 0.01	0.13 ± 0.02	T_(56,2)_=1.5, *p*=.14
Left NAcc	0.05 ± 0.01	0.05 ± 0.01	T_(56,2)_=0.4, *p*=.69
Right HC	0.29 ± 0.03	0.30 ± 0.03	T_(56,2)_=0.5, *p*=.65
Right AMY	0.12 ± 0.01	0.12 ± 0.02	T_(56,2)_=0.9, *p*=.38
Right NAcc	0.05 ± 0.01	0.05 ± 0.01	T_(56,2)_=1.3, *p*=.20
Right ACC	0.39 ± 0.05	0.39 ± 0.05	T_(56,2)_=0.6, *p*=.53
Right IFG_op_	0.25 ± 0.04	0.24 ± 0.03	T_(56,2)_=1.0, *p*=.34
Right IFG_orb_	0.07 ± 0.01	0.07 ± 0.02	T_(56,2)_=0.3, *p*=.73
Right IFG_tri_	0.19 ± 0.04	0.19 ± 0.04	T_(56,2)_=0.1, *p*=.94
Right MFG	0.72 ± 0.14	0.66 ± 0.14	T_(56,2)_=1.6, *p*=.11
Left ACC	0.36 ± 0.04	0.35 ± 0.05	T_(56,2)_=0.7, *p*=.50
Left IFG_op_	0.27 ± 0.05	0.26 ± 0.05	T_(56,2)_=0.6, *p*=.54
Left IFG_orb_	0.07 ± 0.02	0.07 ± 0.02	T_(56,2)_=0.3, *p*=.80
Left IFG_tri_	0.22 ± 0.05	0.21 ± 0.05	T_(56,2)_=0.8, *p*=.44
Left MFG	0.81 ± 0.14	0.76 ± 0.15	T_(56,2)_=1.5, *p*=.13

Note

Abbreviations: ADHD, attention‐deficit/hyperactivity disorder, TDC, typically developing children, HC, hippocampus, AMY, amygdala, NAcc, nucleus accumbens, ACC, anterior cingulate cortex, IFG, inferior frontal gyrus, IFG_op_, opercular part of the IFG, IFG_orb_, orbital part of the IFG, IFG_tri_, triangular part of the IFG, MFG, middle frontal gyrus; FDR correction for 27 comparisons revealed a q* = 0.002.

### 3.1 Reverse *Tph2^‐/‐^* phenotype

Impulsivity positively correlated with aggression, but not anxiety. Aggression and anxiety were negatively related; all correlations were significant across all subjects and genotype‐specifically (impulsivity*aggression: *R *= .541, *p *= .000; R_GG_ **= 0.420, *p *= .015, R_T+_ **= 0.582, *p *= .002; Z Z_GGvs.T+_ **= 1.1, *p *= .149; aggression*anxiety: *R *= −.378, *p *= .003; R_GG_ **= −0.341, *p *= .052, R_T+_ **= −0.408, *p *= .043, Z_GGvs.T+_ **= 0.4, *p *= .356). Neither impulsivity, nor aggression, or anxiety differed significantly between sexes, and only anxiety correlated with age (*R*_impulsivity_ **= −0.181, *p *= .086; *R*_aggression_ **= −0.238, *p *= .065; *R*_anxiety_ **= 0.356, *p *= .001).

#### 3.1.1 Analysis step 1

Group comparisons did not reveal any significant difference between *TPH*2 *G‐703T* variants; however, in absolute values T^+^ were more impulsive compared with GG (uncorrected *p *< .05), and aggression scores were higher (Table 1).

#### 3.1.2 Analysis step 2a

**FIGURE 1:**
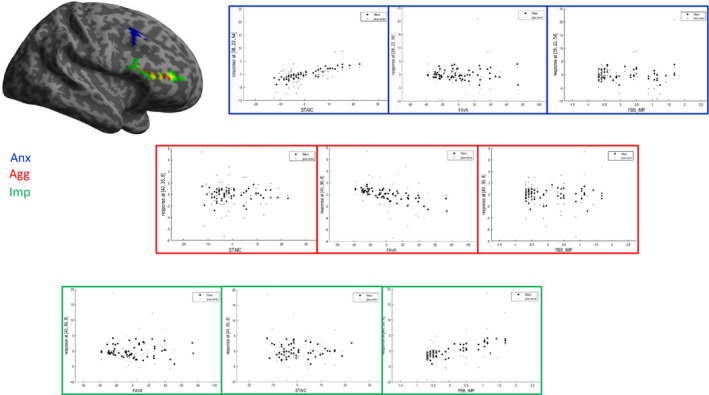
represents brain regions, associated with the *reversed Tph2^‐/‐^ phenotype*. On the left, brain activation is plotted on a representative brain surface. On the right, scatterplot represents correlations

Multiple regressions revealed (a) on the behavioral level, a positive influence of impulsivity on rt (Table S2). (b) On brain structure, no significant correlations were found (Table S3). (c) In brain activation, the *reversed Tph2^‐/‐^ phenotype* correlated predominantly with activation in the reward condition: Whereas impulsivity and aggression correlated with overlapping regions in the triangular part of the right IFG and the right MFG, anxiety correlated with an isolated region in right MFG. In addition, correlation with aggression was negative, and correlations with impulsivity and anxiety were positive (Table 2, Figure 1). During cue processing, impulsivity and aggression positively correlated with activation in the opercular part of the IFG (impulsivity: left IFG, aggression: right IFG), the same was found for anxiety during target processing (right IFG) (Table 2).

#### 3.1.3 Analysis step 2b

Mediation analyses using (a) behavioral, (b) structural, and (c) functional parameters alone did not reveal a significant interaction with *TPH*2 *G‐703T* and the impulsive/aggressive/anxious phenotype.

**FIGURE 2:**
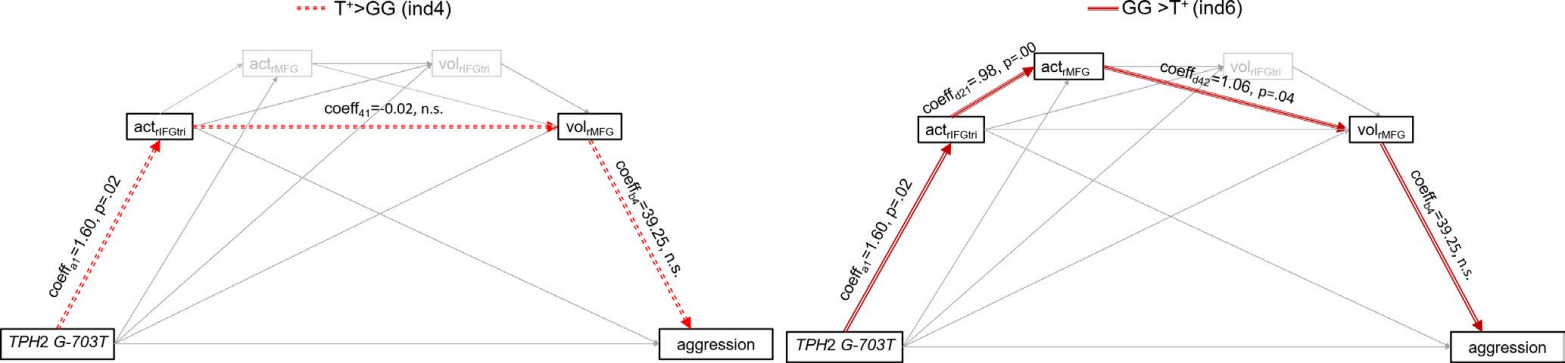
represents the significant mediation model for aggression as part of the *reversed Tph2^‐/‐^ phenotype* with the independent factor X = *TPH*2 *G‐703T*, the dependent variable Y = aggression, and mediating variables M1 = aggression‐related activation in the right IFG_tri_, M2 = aggression‐associated activation in the MFG, M3 = right IFG_tri_ volume, and M4 = right MFG volume. On the left side, significant T+‐specific indirect effect is presented, that is, in T‐allele carriers, the *TPH*2 *G‐703T* genotype is related to trait aggression not directly but via right IFG activation and right MFG volume. On the right side, GG‐specific indirect effect is depicted, that is, in GG homozygotes, and the *TPH*2 *G‐703T* genotype is related to trait aggression not directly but via right IFG activation, MFG activation and right MFG volume

However, when combining structural and functional parameters, significant serial mediations were found: The relation between the *TPH*2 *G‐703T* genotype and the aggressive phenotype was mediated by brain activation in the right IFG (triangular part) and right MFG volume (Table 3, ind 4), and right IFG volume (Table 3, ind 6). Furthermore, indirect effects differed between genotypes: Whereas the mediation effect of IFG (triangular part) activation and MFG volume (ind 4) was stronger in T^+^, mediation effect of IFG (triangular part) activation, MFG activation, and MFG volume (ind 6) was stronger in GG (Figure 2).

## 4 DISCUSSION

In this study, we addressed the mechanisms underlying the *reversed Tph2^‐/‐^ phenotype* in children and adolescents with and without ADHD. We showed that the influence of *TPH*2 *G‐703T* on aggression (as part of the *reversed Tph2^‐/‐^ phenotype*) was mediated by brain activation and regional volume of the triangular part of the right IFG and the right MFG. We did not find similar associations for impulsivity and anxiety. Thus, for aggression we were able to demonstrate a similar genotype/phenotype relation in mice and humans.

### 4.1 Aggression and prefrontal structures

Aggression in humans is associated with an imbalance within a cortico‐limbic network (Rosell & Siever, 2015), that is, a deficient regulation of the amygdala via prefrontal areas (Coccaro et al., 2011). Fronto‐amygdala pathways have been involved in the control of aggressive impulses (Bufkin & Luttrell, 2005); reduced volumes in the prefrontal cortex and amygdala were associated with antisocial traits (Gregory et al., 2012), MFG activation counteracted with aggressive reactions (Achterberg et al., 2016), and IFG–amygdala connectivity was reduced in aggressive individuals (Bogerts et al., 2018). Specifically, findings suggested right‐lateralized alterations for both volume reductions in the prefrontal cortex (Cha et al., 2015) and IFG–amygdala connectivity (Gilam et al., 2017). In line with these findings, we found that prefrontal regions were lateralized in the right hemisphere and that predominantly the IFG and MFG were involved.

However, we did not find any correlation between aggression and amygdala. This finding could not be explained by diagnostic group, as (a) all analyses were corrected for group and (b) there were no a priori group differences between ADHD and TDC in our sample. In addition, post hoc group‐specific multiple regressions did not reveal significant relations between aggression and amygdala volume neither for ADHD nor for TDC (ADHD: beta_aggression_ **= 0.06, *p *= .792; TDC: beta_aggression_ **= 0.04, *p *= .858). Likewise, the lack of relation between aggression and amygdala volume was not confounded by group‐specific brain development, as multiple regressions were corrected for age, and post hoc group‐specific age correlations did not reveal a significant correlation (ADHD: R_rAMY_vol*age_ **= −0.07, *p *= .722; TDC: R_rAMY_vol*age_ **= −0.02, *p *= .921). Thus, especially the role of the amygdala should be further investigated in future studies including greater sample sizes.

### 4.2 Aggression and TPH2 G‐703T

Aggression has been shown to vary between *TPH*2 *G‐703T* variants in numerous studies. For example, Yoon et al. (2012) linked *TPH*2 *G‐703T* to anger‐related personality traits, finding that GG had a higher anger control scores compared with T^+^ and that the correlation between aggression and orbitofrontal cortex volume differed between genotypes, suggesting that the orbitofrontal cortex is “an intermediate phenotype that bridges serotonin synthesis and anger‐related traits. The mechanism underlying the effect of the *TPH*2 gene on OFC abnormality, however, may be complex and may involve several processes related to anger expression.” (Yoon et al., 2012) More generally, Wolf et al. (2018) showed serotonergic modulation of aggression in a pharmacological fMRI study. Participants were playing a video game during fMRI scanning after a single dose of selective 5‐HT reuptake inhibitor (escitalopram). They found that medication reduced neural response in the right IFG and ACC to violent but not nonviolent actions and underlined the validity of serotonin in the modeling of aggressive behavior (Rosell & Siever, 2015; Wolf et al., 2018). Similar effects have also been found in mice: After injections of 5‐HT receptor agonists in the prefrontal cortex, animals showed reduced aggressive behavior in terms of attack bites and lateral threats (Centenaro et al., 2008). Likewise, we found that the mediation effect differed between *TPH*2 *G‐703T* variants: In contrast to GG, in T^+^ IFG volume was not a significant mediator. Thus, our results expand on these findings, (a) suggesting IFG as further region increasing susceptibility to serotonergic imbalance and (b) showing that brain function in addition to structure plays a role in serotonergic modulation.

### 4.3 Relation between impulsivity, aggression, and anxiety

At first sight, examining impulsivity, aggression, and anxiety in humans may seem counter‐intuitive (Cohn et al., 2016). However, they represent a cohesive behavioral program for reacting in stressful and dangerous situations: the *fight or flight response*, with *fight* representing the “impulsive/aggressive” way to react and *flight* the rather “anxious” manner to respond. Thus, impulsivity, aggression, and anxiety represent internal emotional states and are natural adaptive consequences of stress to cope with the stressor (Lesch, 2005). Additionally, in the context of ADHD symptoms aggression and anxiety have been shown to closely interact (Schatz & Rostain, 2006), even though the direction of relations is still being discussed. The exacerbation hypothesis proposes that the presence of anxiety increases the risk of aggression arguing that anxiety increases the emotional response to a stressful situation, thus, lowers the threshold for the need to react, and inhibits the fine‐tuned behavioral reaction. The attenuation hypothesis assumes that the presence of anxiety protects against aggression as anxiety inhibits process inducing rather freezing behavior than aggressive one. A very recent study, in return, showed that the relation between aggression and anxiety was direct (neither mediated nor moderated by further variables), that is, aggression was negatively related to anxiety and did not interact with ADHD symptoms at all (Murray et al., 2018). Overlapping function between impulsivity and aggression and inverse relation between aggression and anxiety could be found in our fMRI findings as well: a priori correlations revealed a positive relation between impulsivity and aggression, whereas it was negative between aggression and anxiety. Likewise, impulsivity‐ and aggression‐related regions were in overlapping areas, whereas anxiety was associated with an isolated region in the MFG. Finally, while the more anxious subjects were, the stronger they activated the MFG, it was reverse in aggression (i.e., the more aggressive subjects were, the weaker MFG and IFG activation).

### 4.4 TPH2 G‐703T (rs4570625) polymorphism and ADHD

Even though numerous papers showed that this polymorphism plays a significant role in the context of ADHD via reduced serotonin in T‐allele carriers, these findings are not undisputed as they were not replicated in recent studies. For example, the *TPH*2 *G‐703T* (rs4570625) polymorphism did not show up as a risk gene in large GWAS study (Demontis et al., 2019) and a recent meta‐analysis (Ottenhof et al., 2018) did not show an association with ADHD. In addition, the common noncoding *G‐703T* polymorphisms have not been conclusively shown to alter expression of *TPH2*, and thus, serotonin function: Scheuch et al. (2007) reported that it is more likely the rs11178997 of the human *TPH2* that significantly reduces promoter activity, not rs4570625 (Scheuch et al., 2007). Similar, Heinrich et al. (2017) showed that methylation in the *TPH2*, probe cg14791008 was associated with reward‐based reaction times and ADHD symptoms in ADHD patients (Heinrich et al., 2017). However, a genome‐wide link between genes, cortical surface area, and ADHD has been shown in a recent paper by Gutiérrez et al. (2002). They found for ADHD significant negative genetic correlations between surface area of the MFG and IFG and numerous chromosomes (see Grasby et al., Table S1), for example, between the triangular part of the IFG and chromosome 11q23.1. Chromosome 11q23.1 has been linked to serotonergic 5‐hydroxytryptamine A‐receptor (Gutiérrez et al., 2002; Weiss et al., 1995), which also have both been reported to be relevant in ADHD (Hou et al., 2018; Li et al., 2014; Oades et al., 2008) undermining the role of serotonin in the context of ADHD in general and manifestation of serotonergic modulation in the triangular part of the IFG in particular. Similar genetic correlation analyses with aggression, in return, did not reveal any significant results (Fernàndez‐Castillo & Cormand, 2016; Grasby et al., 2020). Thus, it would be crucial in future studies (a) to examine multiple (*TPH2*) gene variants and/or perform genome‐wide analyses, (b) to quantify their influence on promotor activity in human brain, for example, via the determination of peripheral serotonin levels, and (c) to address multiple behavioral/cognitive dimensions like in the reversed phenotyping approach, when studying *TPH2* effects on brain structure and function of the normally and pathologically developing brain.

## 5 LIMITATIONS

In summary, we examined the *reversed Tph2^‐/‐^ phenotype* was present in humans. We were able to detect a mechanism for aggression but not for impulsivity and anxiety. One argument is that the *reversed Tph2^‐/‐^ phenotype* is a phenotype of a complete knockout mouse, which is not reflected by T^+^. Analogies between heterozygote knockout mice would have been more realistic, and therefore, it is plausible that we did not find the full phenotype. Another explanation might be that the waiting impulsivity network was too complex. Impulsivity tasks stimulating, for example, only the fronto‐striatal loop such as the GoNogo task might have shown similar PFC results, however, leaving more statistical variance to only one further region instead of multiple, enhancing the probability of significant results also for the second region (Cohen, 2013). However, in our study, we chose the 4‐CSRTT as the current study is part of a translational project and the 4‐CSRTT is a task of which versions for both humans and animals have been designed and published by the same laboratory (Bari et al., 2008; Voon et al., 2014), offering a unique chance to compare behavioral performance between two species.

Finally, the approach of mediation analyses has statistical limitations. One major aspect is that mediations promise to predict causal relations. However, this is only the case, if one pre‐assumption is fulfilled, the no confounding or ignorability assumption (Lee et al., 2019). This means that all major variables are implemented in the model. Thus, potential confounders might also have influenced our findings, for example, the bivariate differentiation between genotypes. We know that epigenetic markers modulate the impact of genotypes (Schuebel et al., 2016); thus, further genetic markers such as methylation (Gottschalk & Domschke, 2016) of *TPH*2 *G‐703T* (rs4570625) would have been highly advantageous. Furthermore, we examine the same variables for three different phenotype dimensions. Thus, insignificant results on anxiety and impulsivity may reflect that additional variables were lacking, which was not the case regarding aggression. Finally, we cannot exclude that “unmeasured confounding may still introduce bias even if known confounders have been adjusted for” page 698, (Lee et al., 2019).

## 6 CONCLUSION

In this study, we used the “reverse phenotyping” approach as a potential methodological way to combine findings from animal and human literature. At the example of aggression, we were able to show that the animal phenotype in humans was based on a similar gene X (brain X)–phenotype interaction as described in animal findings. In our view, this is a promising approach for a direct way to examine experimental models in psychiatric populations. Future studies need to confirm how the found relationships may account for ADHD‐associated symptoms and covarying comorbid disorders in the context of ongoing development throughout early and later adulthood.

## CONFLICT OF INTEREST

None of the authors declare any conflict of interest.

## **AUTHOR** **CONTRIBUTION**

KPL, MR, AS‐B, and SN were involved in designing the concept of project; KPL and AS‐B involved in the animal part of the study and genotyping; and MR and SN involved in the human part of the study and funding acquisition. SN and KP were responsible for recruitment and examination of participants (phenotyping and scanning). SN, AA, and AR performed statistical analyses and manuscript drafting. All participants finalized the submitted manuscript.

### PEER REVIEW

The peer review history for this article is available at https://publons.com/publon/10.1002/brb3.2054.

## Supporting information

Fig S1

Table S1

Table S2

Table S3

## Data Availability

The data that support the findings of this study are available from the corresponding author upon request.
