# Supplementary figures and images for: REVERSE phenotyping—Can the phenotype following constitutive Tph2 gene inactivation in mice be transferred to children and adolescents with and without adhd?
Source: Brain Behav. 2021 Feb 1;11(5):e02054. doi: 10.1002/brb3.2054 (PMC8119824; doi:10.1002/brb3.2054)

## Slide 1
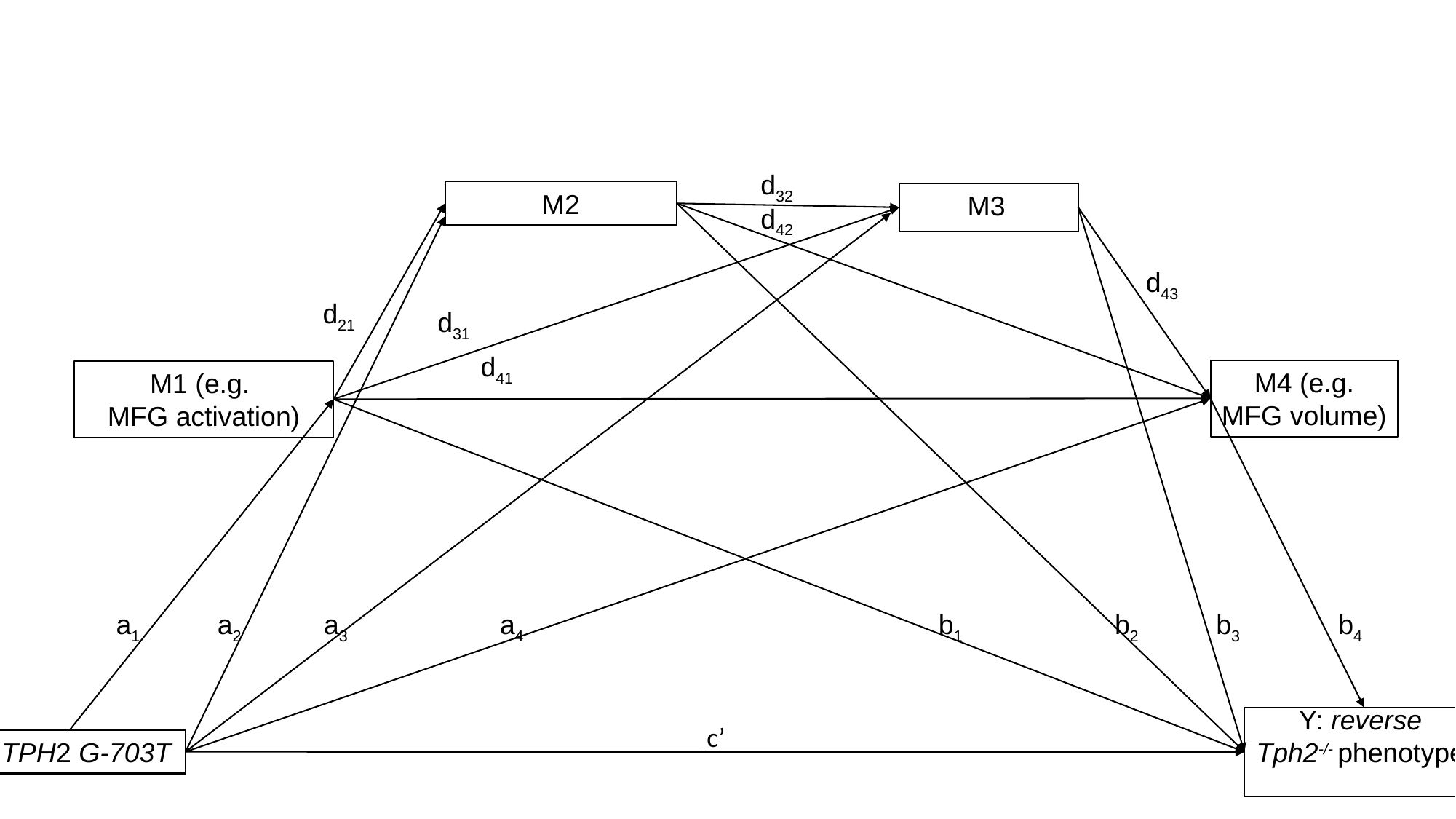

d32
M2
M3
d42
d43
d21
d31
d41
M4 (e.g. MFG volume)
M1 (e.g.
MFG activation)
a1
a2
a3
a4
b1
b2
b3
b4
Y: reverse
Tph2-/- phenotype
c’
X: TPH2 G-703T

Supplement: Supplementary file 1 — Fig S1 [file BRB3-11-e02054-s002.pptx]
