# Supplementary material for: REVERSE phenotyping—Can the phenotype following constitutive Tph2 gene inactivation in mice be transferred to children and adolescents with and without adhd?
Source: Brain Behav. 2021 Feb 1;11(5):e02054. doi: 10.1002/brb3.2054 (PMC8119824; doi:10.1002/brb3.2054)
Supplement: Supplementary file 2 — Table S1 [file BRB3-11-e02054-s001.docx]

**Table s1** group differences between ADHD patients and TDC using ANCOVA Models independent factor diagnostic group (ADHD vs. TDC), and age as nuisance variable

|  | **ADHD** | **TDC** | | **Statistics** |
| --- | --- | --- | --- | --- |
| Sex (m/f) | 29/3 | 23/3 | | *Χ*^2^=.07, p=.56 |
| Age | 12.7±2.2 | 14.5±2.0 | | T_(56,2)_=3.4**, p=.00 |
| IQ | 101.7±11.5 | 109.2±16.6 | | T_(56,2)_=2.0, p=.05 |
| Tanner Stages | 2.6±1.2 | 3.6±1.1 | | T_(56,2)_=3.3**, p=.00 |
| **ADHD symptom severity** | | |  |  |
| FBB_ADHD [inattention] | 1.8^a^ ±0.6 | 0.4^a^±0.3 | | F_(55,3)_=64.7**, p=.00 |
| CBCL [internal problems] | 60^a^.6±7.1 | 49.4^a^±9.6 | | F_(55,3)_=12.7**, p=.00 |
| CBCL [external problems] | 58^a^.1±8.3 | 43.7^a^±8.3 | | F_(55,3)_=21.4**, p=.00 |
| ***reversed Tph2^-/-^ phenotype*** | |  | |  |
| impulsivity (FBB_Imp) | 1.2^a^ ±0.7 | 0.1±0.14 | | F_(55,3)_=29.2**, p=.00 |
| aggression (FAVK) | 50^a^.6±28.8 | 23.5±13.5 | | F_(55,3)_=9.7**, p=.00 |
| anxiety (STAIC-T) | 33^a^.3±8.9 | 31.9±6.9 | | F_(55,3)_=3.3, p=.05 |
| **behavioral performance** | | |  |  |
| premature responses | 2.5^a^±1.5 | 2.9^a^±2.0 | | F_(55,3)_=2.2, p=.12 |
| accuracy [%] | 79.2^a^±14.4 | 81.7^a^±14.9 | | F_(55,3)_=6.8*, p=.00 |
| reaction times [ms] | 467^a^±102 | 428^a^±68 | | F_(55,3)_=14.8**, p=.00 |
| **regional volumes** | | |  |  |
| left HC | .29^a^±.03 | .29^a^±.02 | | F_(55,3)_=0.8, p=.44 |
| left AMY | .12^a^±.02 | .12^a^±.02 | | F_(55,3)_=0.2, p=.73 |
| left NAcc | .05^a^±.01 | .05^a^±.01 | | F_(55,3)_=2.6, p=.09 |
| right HC | .29^a^±.03 | .29^a^±.03 | | F_(55,3)_=0.2, p=.79 |
| right AMY | .12^a^±.01 | .12^a^±.02 | | F_(55,3)_=0.1, p=.87 |
| right NAcc | .05^a^±.01 | .05^a^±.01 | | F_(55,3)_=1.9, p=.16 |
| right ACC | .39^a^±.05 | .39^a^±.06 | | F_(55,3)_=1.1, p=.34 |
| right IFG_op_ | .24^a^±.04 | .24^a^±.03 | | F_(55,3)_=0.1, p=.91 |
| right IFG_orb_ | .07^a^±.01 | .07^a^±.02 | | F_(55,3)_=2.6, p=.08 |
| right IFG_tri_ | .19^a^±.04 | .19^a^±.04 | | F_(55,3)_=0.8, p=.46 |
| right MFG | .67^a^±.15 | .72^a^±.14 | | F_(55,3)_=1.1, p=.33 |
| left ACC | .34^a^±.05 | .37^a^±.05 | | F_(55,3)_=2.1, p=.13 |
| left IFG_op_ | .27^a^±.05 | .26^a^±.04 | | F_(55,3)_=2.9, p=.06 |
| left IFG_orb_ | .07^a^±.02 | .06^a^±.02 | | F_(55,3)_=0.9, p=.40 |
| left IFG_tri_ | .22^a^±.04 | .21^a^±.05 | | F_(55,3)_=1.7, p=.19 |
| left MFG | .76^a^±.14 | .82^a^±.15 | | F_(55,3)_=1.2, p=.31 |

**Note. ^a:^** covariates appearing in the model are evaluated at the values: age=13.5yrs. ADHD: attention-deficit/hyperactivity disorder, TDC: typically developing children, HC: hippocampus, AMY: amygdala, NAcc: nucleus accumbens, ACC: anterior cingulate cortex, IFG: inferior frontal gyrus, IFG_op_: opercular part of the IFG, IFG_orb_: orbital part of the IFG, IFG_tri_: triangular part of the IFG, MFG: middle frontal gyrus; FDR-correction for 30 comparisons revealed a q*=.00125, *: FDR-corrected significant.
