# Supplementary material for: REVERSE phenotyping—Can the phenotype following constitutive Tph2 gene inactivation in mice be transferred to children and adolescents with and without adhd?
Source: Brain Behav. 2021 Feb 1;11(5):e02054. doi: 10.1002/brb3.2054 (PMC8119824; doi:10.1002/brb3.2054)
Supplement: Supplementary file 3 — Table S2 [file BRB3-11-e02054-s004.docx]

**Table s2** multiple regressions of the *reversed* *Tph2^-/-^ phenotype* on behavioral performance using impulsivity, aggression, and anxiety as independent regressors and behavioral parameters as dependent variables. Diagnostic group and age were used as nuisance variables

|  | **F_Model_** | **R^2^** | | **beta_impulsivity_** | | **beta_aggression_** | | **beta_anxiety_** | |  |
| --- | --- | --- | --- | --- | --- | --- | --- | --- | --- | --- |
| **behavioral performance** | | |  | |  | |  | |  | |
| premature responses | 0.1, p=.98 | 0.00 | | -0.04, p=.81 | | 0.08, p=.67 | | -0.02, p=.90 | |  |
| accuracy | 3.4, p=.02 | 0.16 | | -0.18, p=.25 | | -0.24, p=.15 | | 0.27, p=.05 | |  |
| reaction time | 13.1*, p=.00 | 0.42 | | 0.69*, p=.00 | | -0.20, p=.14 | | -0.18, p=.12 | |  |

**Note.** *: FDR-corrected significant q*=.02
