# Supplementary material for: REVERSE phenotyping—Can the phenotype following constitutive Tph2 gene inactivation in mice be transferred to children and adolescents with and without adhd?
Source: Brain Behav. 2021 Feb 1;11(5):e02054. doi: 10.1002/brb3.2054 (PMC8119824; doi:10.1002/brb3.2054)
Supplement: Supplementary file 4 — Table S3 [file BRB3-11-e02054-s003.docx]

**Table s3** multiple regressions of the *reversed* *Tph2^-/-^ phenotype* on regional brain volumes, using impulsivity, aggression, and anxiety as independent regressors and regional brain volumes as dependent variables. Diagnostic group and age were used as nuisance variables

|  | **F_Model_** | **R^2^** | | **beta_impulsivity_** | | **beta_aggression_** | | **beta_anxiety_** | |
| --- | --- | --- | --- | --- | --- | --- | --- | --- | --- |
| **Regional brain volumes** | |  |  | |  | |  | |  |
| left HC | 0.8, p=.51 | .04 | | .16, p=.33 | | -.25, p=.16 | | .02, p=.90 | |
| left AMY | 0.8, p=.50 | .05 | | -.24, p=.15 | | .18, p=.32 | | .01, p=.99 | |
| left NAcc | 0.9, p=.45 | .05 | | -.08, p=.64 | | .15, p=.41 | | .14, p=.35 | |
| right HC | 0.2, p=.89 | .01 | | .11, p=.53 | | -.09, p=.62 | | -.03, p=.85 | |
| right AMY | 0.2, p=.87 | .01 | | -.14, p=.41 | | .09, p=.62 | | -.02, p=.88 | |
| right NAcc | 1.9, p=.14 | .10 | | -.09, p=.57 | | .26, p=.14 | | .15, p=.32 | |
| right ACC | 0.8, p=.51 | .0 | | -.20, p=.22 | | .24, p=.19 | | -.14, p=.35 | |
| right IFG_op_ | 2.6, p=.06 | .13 | | -.04, p=.82 | | .05, p=.78 | | -.38, p=.01 | |
| right IFG_orb_ | 2.1, p=.11 | .11 | | -.17, p=.29 | | .24, p=.17 | | -.36, p=.02 | |
| right IFG_tri_ | 0.1, p=.98 | .01 | | .01, p=.95 | | -.05, p=.79 | | .01, p=.99 | |
| right MFG | 1.1, p=.37 | .06 | | -.27, p=.10 | | .09, p=.60 | | .03, p=.82 | |
| left ACC | 2.3, p=.09 | .12 | | -.29, p=.07 | | -.01, p=.99 | | -.17, p=.24 | |
| left IFG_op_ | 1.0, p=.38 | .06 | | -.12, p=.48 | | .20, p=.27 | | -.26, p=.09 | |
| left IFG_orb_ | 0.6, p=.61 | .04 | | .01, p=.96 | | .10, p=.58 | | -.20, p=.19 | |
| left IFG_tri_ | 1.1, p=.38 | .06 | | .11, p=.51 | | .17, p=.34 | | -.12, p=.44 | |
| left MFG | 0.5, p=.68 | .03 | | -.20, p=.25 | | .06, p=.72 | | -.05, p=.76 | |

**Note.** HC: hippocampus, AMY: amygdala, NAcc: nucleus accumbens, ACC: anterior cingulate cortex, IFG: inferior frontal gyrus, IFG_op_: opercular part of the IFG, IFG_orb_: orbital part of the IFG, IFG_tri_: triangular part of the IFG, MFG: middle frontal gyrus; FDR-correction for 16 comparisons revealed a q*=.0025.
